# Evaluation of a low-intensity pulsed ultrasound for chronic prostatitis type IIIb/chronic pelvic pain syndrome: a randomized controlled trial

**DOI:** 10.3389/frph.2025.1714803

**Published:** 2025-12-04

**Authors:** Jiahao Huang, Zedong Liao, Shankun Zhao, Bodong Lv, Ke Liang

**Affiliations:** 1 Department of Urology, Taizhou Central Hospital (Taizhou University Hospital), Taizhou, Zhejiang, China; 2 Department of Urology, The Second Affiliated Hospital of Zhejiang University School of Medicine, Hangzhou, Zhejiang, China; 3 Department of Urology, The First People’s Hospital of Pinghu, Pinghu, Zhejiang, China

**Keywords:** low-intensity pulsed ultrasound, tamsulosin sustained-release, chronic prostatitis type IIIb/chronic pelvic pain syndrome, efficacy, safety

## Abstract

**Background:**

Chronic prostatitis type IIIb/chronic pelvic pain syndrome (CP/CPPS) poses significant therapeutic challenges. This study aimed to investigate and compare the clinical efficacy and safety of low-intensity pulsed ultrasound (LIPUS) versus tamsulosin for treating CP/CPPS.

**Methods:**

In this randomized controlled trial, 65 patients with CP/CPPS were allocated to two groups. Group A (*n* = 35) received LIPUS treatment twice weekly, while Group B (*n* = 30) received tamsulosin sustained-release capsules (0.2 mg, once nightly). The treatment duration was four weeks for all patients. Outcomes were assessed using the National Institutes of Health Chronic Prostatitis Symptom Index (NIH-CPSI), Self-Rating Anxiety Scale (SAS), and International Index of Erectile Function-5 (IIEF-5) at baseline and 4 weeks post-intervention.

**Results:**

After 4 weeks, both groups showed significant improvements in all NIH-CPSI domains (pain, urinary, quality of life), SAS, and IIEF-5 scores compared to baseline (all *P* < 0.05). Group A demonstrated significantly greater improvement in pain symptoms than Group B (*P* < 0.05), whereas Group B showed superior improvement in urinary symptoms compared to Group A (*P* < 0.05). No statistically significant differences were found between the groups for the remaining symptom domains (*P* > 0.05).

**Conclusion:**

Both LIPUS and tamsulosin significantly alleviated CP/CPPS-related symptoms with a favorable safety profile. LIPUS was more effective for pain relief, while tamsulosin was superior for urinary symptoms. Combination therapy may represent a promising approach for managing CP/CPPS.

## Introduction

Chronic prostatitis/chronic pelvic pain syndrome (CP/CPPS) is a frequently encountered urological condition marked by persistent discomfort in the pelvic region, manifestations of lower urinary tract dysfunction (LUTS), and impairments in sexual function.This condition poses significant clinical challenges due to its high incidence, recalcitrant nature, and frequent recurrence (1). Epidemiological data indicate that CP/CPPS affects an estimated 6%–33% of the male population in China (2). Multifactorial pathogenesis involving subclinical pathogen colonization,neurogenic inflammation, and pelvic floor myofascial dysfunction (3).

Conventional management of CP/CPPS primarily involves pharmacotherapy (tailored to symptomatology and history) supplemented by general measures such as lifestyle modifications. However, suboptimal treatment outcomes and high relapse rates persist as major therapeutic challenges in urological practice (4). Emerging physical modalities, including extracorporeal shockwave therapy and therapeutic ultrasound, have gained increasing attention as potential treatments in recent years (5).

Current first-line pharmacotherapy employs *α*_1_-adrenoceptor antagonists (e.g., tamsulosin) to reduce functional urethral resistance by 40%–60% through selective *α*_1a_-subtype inhibition. However, >50% of patients experience recurrence within 1 year (6). Low-intensity pulsed ultrasound (LIPUS) represents a non-invasive, safe form of microenergy therapy defined by its low acoustic intensity and pulsed emission profile. While LIPUS application for treating mild-to-moderate erectile dysfunction (ED) is maturing-supported by multicenter Chinese clinical trials demonstrating efficacy in improving erectile function and reversing pathological changes in the corpus cavernosum (7), its utility for CP/CPPS requires rigorous validation.

Chronic prostatic inflammation constitutes a key pathological feature of CP/CPPS. Preclinical studies have established LIPUS's anti-inflammatory potential, evidenced by its inhibition of bacterial lipopolysaccharide (LPS)-induced phosphorylation of key protein kinases (ERK, p38 MAPK, Akt) and attenuation of TLR4 signal transduction in target cells (8). This mechanistic insight provides a sound rationale for exploring LIPUS in CP/CPPS. Consequently, this randomized controlled trial seeks to evaluate and contrast the therapeutic effectiveness and safety of LIPUS in comparison with tamsulosin sustained-release capsules among individuals diagnosed with CP/CPPS, primarily aiming to alleviate and manage symptoms associated with this condition.

## Materials and methods

### Study population

A total of 65 patients with NIH Category III CP/CPPS were consecutively enrolled from the Department of Urology, the Second Affiliated Hospital of Zhejiang University School of Medicine and Pinghu First People's Hospital between January 2022 and December 2024, aged 18–56 years.

### Selection criteria

Inclusion criteria required: (i) diagnosis of CP/CPPS per NIH classification with persistent clinical symptoms for >3 months, (ii) absence of concurrent therapies (e.g., antibiotics, extracorporeal shockwave treatment) during the intervention period, (iii) provision of written informed consent.

Exclusion criteria required: (i) documented hypersensitivity to tamsulosin sustained-release or renal impairment, (ii) confounding urological conditions: benign prostatic hyperplasia, active urinary tract infections, neurogenic bladder, or urolithiasis, (iii) perineal anatomical abnormalities or prior pelvic surgery/radiotherapy, (iv) diagnosed coagulopathies or thrombotic disorders.

### Study protocol

According to the inclusion criteria, 65 patients with CP/CPPS were recruited from outpatient clinics and randomly allocated to two groups: Group A received LIPUS (twice weekly for four weeks), while Group B was administered tamsulosin hydrochloride (0.2 mg sustained-release capsules orally once daily at bedtime for four weeks). All patients documented NIH-CPSI scores, SAS scores, IIEF-5 scores, and recorded adverse events before and after the 4-week intervention.

### LIPUS therapy application

Transperineal LIPUS therapy was delivered using a Y-shaped (circular) probe (WBL-ED device, Beijing WBL Medical Equipment Co). The initial treatment session employed an intensity of 300 mW/cm² and escalated to 360 mW/cm² (sessions 2–8) to mitigate treatment adaptation. Patients were positioned supine with hips abducted and flexed. The probe was oriented perpendicular to the perineal body midpoint (2 cm anterior to anus, 1 cm posterior to scrotum) with ultrasound gel.Each session lasted approximately 20 min, administered twice weekly (with 1–2-day intervals) over four weeks, totaling eight sessions.

### Statistical analysis

GraphPad Prism software (Version 10.0.2) was used for all statistical analyses. Data normality was evaluated using the Shapiro–Wilk test. Continuous variables conforming to normal distribution were reported as mean ± SD. Pre-post changes within each group​ were examined with paired t-tests. A two-sided *P* < 0.05 indicated statistical significance. All continuous variables not following a normal distribution were presented as median (25th percentile, 75th percentile). Where normality or homogeneity of variance assumptions were violated, non-parametric tests (Wilcoxon signed-rank for intragroup) were implemented at *P* < 0.05.

### Ethics approval and consent to participate

This study was approved by the Institutional Review Boards (IRB) of the Second Affiliated Hospital of Zhejiang University School of Medicine (approval no. 20220367) and The First People's Hospital of Pinghu (approval no. 2023088). Written informed consent was obtained from all participants prior to enrollment. The trial was registered with the Chinese National Medical Research Registration Filing Information System (registration number: MR-33-25-055872).

## Results

### Patients' information

A total of 65 patients were enrolled, The mean age of the patients was 34.36 ± 9.04 years (range: 18–56 years), The mean disease duration was 12.64 ± 4.56 months (range: 3–26 months).No statistically significant differences in baseline characteristics (including age, disease duration) were observed between the intervention and control groups (*P* > 0.05), confirming cohort comparability at enrollment (Table 1).

**Table 1:** Baseline characteristics of the study population at randomization (*n* = 65).

Group	Total	Group A	Group B	*P*-value
*N*	65	35	30	
Ages (years)	34.35 ± 9.11	34.37 ± 9.81	34.33 ± 8.38	0.980
Course of CP/CPPS (months)	12.65 ± 4.59	12.31 ± 5.26	13.03 ± 3.72	0.522

### NIH-CPSI scores

No significant intergroup difference in baseline NIH-CPSI scores was observed before intervention. After 4 weeks of treatment, both groups exhibited statistically significant improvements in all NIH-CPSI domains (pain, urinary, and quality of life (QoL); *P* < 0.05). Notably, Group A demonstrated significantly greater improvement in pain domains than Group B, whereas Group B showed superior amelioration in urinary domains compared to Group A (Table 2).

**Table 2:** Various scores of the two groups of patients before and after treatment.

Group	A			B		
*N*	35			30		
	Baseline	Post-treatment	*P*-value	Baseline	Post-treatment	*P*-value
NIH-CPSI	24.26 ± 7.54	14.46 ± 4.27	<0.05	24.93 ± 7.03	15.53 ± 5.36	<0.05
SAS	39.54 ± 8.57	33.43 ± 5.60	<0.05	40.20 ± 8.48	34.10 ± 4.75	<0.05
IIEF-5	21.00 (15.00, 23.00)	23.00 (20.00, 25.00)	<0.05	21.50 (16.75, 24.00)	23.50 (19.75, 25.00)	<0.05
Pain	11.26 ± 4.99	5.60 ± 2.63	<0.05	11.47 ± 5.19	7.83 ± 4.22*	<0.05
Urination	4.80 ± 2.39	3.23 ± 1.77	<0.05	4.93 ± 2.53	2.13 ± 1.48*	<0.05
Quality	8.20 ± 2.42	5.63 ± 1.83	<0.05	8.53 ± 2.47	5.57 ± 1.61	<0.05

Non-parametric tests were used for the indicators of the IIEF-5, while paired t-tests were used for all other indicators.

* *P* < 0.05, compared with Group A.

### IIEF-5 scores

After 4 weeks of treatment, both groups exhibited statistically significant increases in IIEF-5 scores from baseline (*P* < 0.05). No significant intergroup difference in IIEF-5 scores improvement was observed (*P* > 0.05) (Table 2).

### SAS scores

Post-treatment SAS scores showed significant reductions from baseline in both groups (*P* < 0.05). The magnitude of SAS score reduction was comparable between groups, with no statistical significance (*P* > 0.05) (Table 2).

### Safety

Two adverse events (AEs) occurred in Group A, transient perineal pain during initial treatment in two cases, both self-resolved without intervention, One AE of mild dizziness occurred in Group B and resolved after rest, None of the three adverse events interrupted the treatment course.

## Discussion

CP/CPPS poses a significant clinical challenge in urology, attributable to its complex pathophysiology and the limited efficacy of existing therapies that primarily target symptom alleviation and quality-of-life enhancement (4, 9). Among pharmacotherapeutic options, antibiotic use remains contentious, with contemporary guidelines predominantly contraindicating their administration in non-inflammatory CP/CPPS (1, 10). In contrast, *α*-adrenergic receptor antagonists, such as tamsulosin, constitute a cornerstone for managing LUTS (11). Concurrently, LIPUS has emerged as a promising modality within “microenergy medicine”.LIPUS is characterized by minimal thermal effects and reliance on non-thermal bioeffects—acoustic cavitation, acoustic streaming, and enhanced mass transfer—to drive mechanotransduction-mediated biological responses (12). These bioeffects collectively suppress inflammation through inhibition of lipopolysaccharide-induced phosphorylation of ERK/p38/Akt kinases and TLR4 signaling, concomitantly regulating matrix metalloproteinase (MMP) activity, promoting stem cell differentiation, and stimulating neurovascular regeneration (8, 13–15). Nevertheless, clinical validation is constrained, as evidenced by Li et al.'s randomized trial involving 60 patients with comorbid ED and CP/CPPS (16). Against this backdrop of unmet clinical needs and evolving therapeutic concepts, we conducted the present randomized controlled trial to evaluate the efficacy of LIPUS vs. the standard pharmacologic agent, tamsulosin.

This randomized controlled trial enrolled 65 patients with CP/CPPS. Both four-week therapeutic regimens—LIPUS and tamsulosin sustained-release—significantly improved pain, urinary, QoL, SAS scores and IIEF-5 scores compared to baseline (all *P* < 0.05).The key finding of this study is the distinct therapeutic profile of each intervention: while both modalities significantly improved overall symptoms, tamsulosin demonstrated superior efficacy in alleviating urinary symptoms (*P* < 0.05), whereas LIPUS was significantly more effective in reducing pelvic pain (*P* < 0.05). This differential effect suggests that these treatments may target distinct pathophysiological pathways underlying CP/CPPS.

The superior improvement in urinary symptoms observed with tamsulosin aligns with established clinical practice and pharmacological principles (11). As a selective *α*_1_-adrenergic receptor antagonist, tamsulosin primarily acts by relaxing smooth muscle in the prostate and bladder neck, thereby reducing dynamic obstruction and improving urinary flow (6). Our findings thus reinforce the role of alpha-blockers as a cornerstone for managing LUTS in CP/CPPS patients, consistent with current guideline recommendations (1, 11).

Within urology, LIPUS has demonstrated therapeutic efficacy for ED as evidenced by reversal of cavernosal pathology in multicenter randomized trials (7). In our study, we demonstrated that LIPUS treatment resulted in significant pain relief, which aligns with previous mechanistic studies indicating that LIPUS may alleviate inflammation and reduce pain by downregulating inflammatory mediators such as COX-2 and IL-1β (6, 16, 17).

Both therapies yielded comparable improvements in QoL, anxiety (SAS), and ED (IIEF-5). This suggests that benefits in these secondary domains may be contingent upon the alleviation of core symptoms, namely pain and urinary disturbances (18, 19). The strong comorbidity between pain, anxiety, and sexual health in CP/CPPS has been substantiated by recent epidemiological studies (20, 21). Consequently, our results reinforce the validity of the biopsychosocial model and the UPOINT phenotyping system, which advocate for a multidimensional assessment and tailored management strategy (22). The incorporation of SAS and IIEF-5 scales in our trial aligns with this modern, patient-centered approach, allowing for a comprehensive evaluation of treatment efficacy beyond core urological symptoms.

This prospective dual-center clinical trial investigated LIPUS therapy for CP/CPPS. Despite its randomized controlled design, limitations include prolonged intervention durations, modest cohort size (*n* = 65), and inadequate longitudinal monitoring (>6 months), which may introduce selection bias and limit generalizability. Consequently, validation via large-scale, multi-center randomized trials is imperative. The data suggest the potential for the combination of LIPUS and tamsulosin sustained-release to offer improved management of CP/CPPS symptoms (pain and urinary). Furthermore, optimization of LIPUS treatment protocols—specifically energy intensity, session duration, and therapeutic scheduling—may potentiate LIPUS-mediated anti-inflammatory and neuromodulatory effects, thereby enhancing symptom management and quality-of-life improvement. However, rigorously designed randomized controlled trials are warranted to validate the long-term efficacy and safety of this combined approach.

## Conclusion

LIPUS and tamsulosin sustained-release capsules exhibit significant clinical efficacy in ameliorating CP/CPPS-related symptoms, with both interventions demonstrating favorable safety profiles. LIPUS was more effective for pain relief, while tamsulosin showed greater benefit for LUTS.

## Data Availability

The original contributions presented in the study are included in the article/Supplementary Material, further inquiries can be directed to the corresponding authors.
